# New data on jumping spiders of the subfamily Spartaeinae (Araneae, Salticidae) from New Guinea, with the description of a new species

**DOI:** 10.3897/zookeys.1286.172848

**Published:** 2026-07-30

**Authors:** Hunor Takács-Vágó, Tamás Szűts, Krisztián Szabó

**Affiliations:** 1 Diversity and Evolution Research group, Department of Zoology, University of Veterinary Medicine, Budapest, Hungary Diversity and Evolution Research group, Department of Zoology, University of Veterinary Medicine Budapest Hungary

**Keywords:** *
Cocalodes
*, *
Gelotia
*, lectotype designation, taxonomy, Wallacea

## Abstract

We describe a new species of *Gelotia* Thorell, 1890, *Gelotia korowai***sp. nov**., which is the first *Gelotia* species from the island of New Guinea. New records for four *Cocalodes* species—*C. innotabilis* Wanless, 1982; *C. macellus* (Thorell, 1878); *C. papuanus* Simon, 1900, and *C. thoracicus* Szombathy, 1915­—are presented, and the specimens are illustrated. Lectotypes for *C. plebejus* Szombathy, 1915 and *C. thoracicus* are also designated. With 49 original images and a distribution map.

## Introduction

Jumping spiders are the most diverse spider family, containing an eighth of all spider species (World Spider Catalog 2026). Studies on the taxonomy of jumping spiders are increasing, and 1,200 species have been described in the past decade, with most (846 spp.) in the past five years (World Spider Catalog 2026).

Recently, Zhang et al. (2024) resolved the phylogenetic placement of Eupoinae (Araneae, Salticidae), which was the last remaining puzzle among higher-level relationships of the subfamilies of Salticidae. Thus, the family is now divided into two main groups. Group 1 contains the subfamilies Salticinae, Onomastinae and Hisponinae, and group 2 contains the subfamilies Spartaeinae, Lyssomaninae, Asemoninae and Eupoinae (Zhang et al. 2024; Ni et al. 2025b). The subfamily Salticinae contains about 93% of the jumping spider diversity (Maddison 2015). Not surprisingly, most new species described are from this group.

The other subfamilies together, often called “basal salticids”, are less speciose than Salticinae. The largest among them, Spartaeinae, contains 224 species in 32 genera. This is ever increasing, as Maddison (2015) reported only 165 species in 29 genera, and this increase in the number of species over a decade is significant. However, only four new genera were described during this time: *Amilaps* Maddison, 2019; *Waymadda* Szűts, Zhang, Gallé-Szpisjak & De Bakker, 2020; *Calxattus* Wang, Yu & Zhang, 2023; and *Protaeus* Ni, Yu & Zhang, 2025. Within Spartaeinae, three tribes have been classified (Maddison 2015), each with a limited distribution: Spartaeini is mostly found in Africa and Asia, with only a few species in Australasia and Europe (Maddison 2015; Metzner 2026); Cocalodini occurs in Australasia (Maddison 2015; Metzner 2026); and Lapsiini is restricted to the New World (Maddison 2015; Metzner 2026). The tribe Spartaeini was divided into two subtribes, Spartaeina (having the tegular furrow) and Holcolaetina (lacking the tegular furrow and possessing a median apophysis and a conductor). However, *Calxattus*, which lacks the tegular furrow and possesses a conductor, has been placed in Spartaeina (by phylogenomic analyses of Ni et al. 2025b, which renders Holcolaetina paraphyletic and, thus, makes division of subtribes problematic; W. Maddison pers. comm.).

While Spartaeini is restricted to the tropics and subtropics of the Old World (Maddison 2015), Cocalodini is strictly Australasian (east of the Wallace Line) and has no clear morphological evidence for monophyly. Molecular data suggest that the Cocalodini are monophyletic, and the subtribe contains the only salticids with a median apophysis east of the Wallace Line (Maddison et al. 2014; Maddison 2015). Cocalodini are common components of New Guinea fauna (Maddison and Zhang 2011; Maddison 2015), with the greatest diversity on the island (Maddison 2009). Before 2008, only two genera and 14 species were known from the tribe, but during the Upper Strickland Expedition, three new genera and six new species were discovered in New Guinea (Maddison 2009). Eleven years later, a fourth genus in the tribe was discovered (Szűts et al. 2020) from Mount Wilhelm, showing that the diversity of the tribe is still underestimated.

In the last decade, no new species have been described from eight genera of Spartaeini, probably due to insufficient exploration of areas like New Guinea, China, and Malaysia (Ni et al. 2025a). In genera such as *Gelotia* or *Portia*, almost half of species are known from a single sex and/or a few specimens (World Spider Catalog 2026). This is likely due to their cryptic appearance (Ni et al. 2025a). The same is true for species of *Cocalodes* in the tribe Cocalodini; all species of this genus were primarily described with limited data from the late 20^th^ century, including a few from the early 1980s (World Spider Catalog 2026) with many descriptions based only on a few drawings and images (Metzner 2026).

In this study, we add to the knowledge of Spartaenini and Cocalodini and describe *Gelotia korowai* sp. nov. from mainland New Guinea, which is the first species of the genus recorded from the island. We also present digital images and new data for four previously described *Cocalodes* species: *C. innotabilis* Wanless, 1982; *C. macellus* Thorell, 1878; *C. papuanus* Simon, 1900, and *C. thoracicus* Szombathy, 1915. We also figure the type series and designate lectotypes for *C. thoracicus* and *C. plebejus* Szombathy, 1915.

## Materials and methods

Types of *Gelotia korowai* are deposited in the Bishop Museum, Honolulu, Hawai‘i, USA. (curator: Adrienne Antonsen). *Cocalodes* specimens are deposited in the Soil Zoology Collections, Hungarian Natural History Museum (curator: Edit Horváth). Specimens were kept in 70% alcohol and were observed under stereomicroscopes. Images were obtained with a Tucsen MiChrome and a Tucsen TreuChrome metrics camera attached to a Nikon SMZ800, a Nikon SMZ1000 and a Nikon Eclipse E200 microscope. Mosaic v. 2.4 (Tucsen Photonics 2022) was used to capture stack images, which were combined into a single multifocal image with Helicon Focus v. 8. Male palps and female genitalia were dissected from the bodies and independently photographed. Female genitalia were placed in methyl-salicylate to make sclerotized parts more transparent (Szűts et al. 2020). Male palps were submerged and photographed in hand sanitizer.

Terminology for *Gelotia* follows Patoleta and Żabka (2020). Leg spination terminology follows Ono (1988). All measurements are given in millimetres. All colour references are from alcohol-preserved specimens. Measurements were obtained with ImageJ v. 1.53t (Schneider et al. 2012). Total length was measured from the anterior margin of the prosoma to the posterior margin of the opisthosoma. Measurements of carapace length were taken from the anterior margin to the posterior margin of the prosoma.

### Abbreviations

**AMNH** American Museum of Natural History, New York, NY, USA;

**BM** Bishop Museum, Honolulu, HI, USA;

**FS** Senckenberg Nature Museum, Frankfurt am Main, Germany;

**HNHM** Hungarian Natural History Museum, Budapest, Hungary;

**MCSN** Museo Civico di Storia Naturale “Giacomo Doria”, Genoa, Italy;

**MNHN** Muséum national d’Histoire naturelle, Paris, France;

**RTA** retrolateral tibial apophysis;

**adt** anterodorsal tubercle (sensu Wanless 1984);

**dta** dorsal tibial apophysis (sensu Patoleta and Żabka 2020);

**vta** ventral tibial apophysis (sensu Patoleta and Żabka 2020);

**dco** dorsal cymbial outgrowth (sensu Patoleta and Żabka 2020);

**pco** prolateral cymbial outgrowth (sensu Patoleta and Żabka 2020);

**rco** retrolateral cymbial outgrowth (sensu Patoleta and Żabka 2020);

**Fe** femur;

**Pa** patella;

**Ti** tibia;

**Mt** metatarsus;

**ap** apical;

**d** dorsal;

**pr** prolateral;

**rt** retrolateral;

**v** ventral.

## Results

### Taxonomy

**Tribe Spartaeini Wanless, 1984**

#### Genus Gelotia

Taxon classificationAnimaliaAraneaeSalticidae

Thorell, 1890

24C0D8DA-74A5-5707-B323-9E71A9D9E390

##### Type species.

*Gelotia frenata* Thorell, 1890 by subsequent designation (Thorell 1892).

##### Diagnosis

(modified from Wanless 1984). Medium-sized spartaeines; highest point of carapace around PLEs (Fig. 2A–C). Male palpal bulb with apical lobe-like part (Fig. 3A–D) from where inward curving embolus originates (Fig. 3A, B). Male palpal tibia with a large retrolateral apophysis (RTA); RTA with thick base and thin apical lamellar part, giving a cap-like appearance to apophysis in ventral and dorsal aspects (Fig. 3A–E; Wanless 1984: figs 17D, 18D, F, 19D, F, 20C, I). Epigyne with thin median ridge (Fig. 4A, Wanless 1984: figs 16F, 17C) and relatively thick copulatory duct (these were interpreted, probably incorrectly, by Wanless (1984: fig. 21C) as a second pair of spermathecae).

##### Description.

For a detailed description, see Wanless (1984).

##### Distribution.

The genus contains 11 species described from East and South Asia, the Indonesian archipelago, Peninsular Malaysia, and New Guinea (Wang and Li 2020; Metzner 2026; World Spider Catalog 2026). All species are endemic to a single country, except for *G. syringopalpis* Wanless, 1984 and *G. robusta* Wanless, 1984; the former is found in China, Borneo, and Malaysia, and the latter in New Britain and Australia (Metzner 2026; World Spider Catalog 2026).

#### Gelotia korowai sp. nov.

Taxon classificationAnimaliaAraneaeSalticidae

F3D829CC-5C28-5A36-9492-BB7D4DDFEB20

https://zoobank.org/4FED67E5-0FEF-41F6-AE1B-FE9B321A49FF

Figs 1, 2, 3, 4

##### Material examined.

***Holotype* male**: Papua New Guinea; • Mt Missim, 6 km N, 7 km W of Wau Aerodrome; 7°17.28'S, 146°39.12'E; 1350 m a.s.l.; 25 August 1988; leg. A. Allison; Tree 21 Tray 7; BM. ***Paratypes***: • 1 male; same data as holotype; Tree 23 Tray 6 • 1 male; same data as holotype; Tree 21 Tray 30 • 1 male; same data as holotype; 20 August 1988; Tree 14 Tray 22. (BM).

##### Etymology.

Named after the Korowai people of New Guinea, who built houses in the canopy. *Gelotia korowai* sp. nov. has been captured by canopy fogging, indicating it also lives in the high canopy. The species name is a noun in apposition.

##### Diagnosis.

Male *Gelotia korowai* sp. nov. have an RTA that is similar to that of *G. robusta* and *G. salax* (Thorell, 1877). It can be easily differentiated from *G. robusta* by the length and width of the embolus, which is thin and long in *G. korowai* sp. nov. (Fig. 3) and short and thick in *G. robusta* (Wanless 1984: fig. 19). It can be easily differentiated from *G. salax* by the oval cymbium (Fig. 3A, B). The retrolateral cymbial outgrowth of *G. salax* is visible above the RTA in ventral view (compare Wanless 1984 fig. 20I with Fig. 3)

As illustrated by Patoleta and Żabka (2020: fig. 8), the embolus is similar in width to that of *G. robusta* but ends at the upper part of the bulb. In *G. korowai* sp. nov., the embolus reaches nearly half the length of the cymbium (Fig. 3B, C, H). Additional differences from *G. robusta*: rco (retrolateral cymbial outgrowth; Fig. 3H) covered by the bulb (Fig. 3H), visible in *G. robusta* (Patoleta and Żabka 2020: fig. 8), the pco (prolateral cymbial outgrowth; Fig. 3F) is a two-pronged cymbial extension (Fig. 3F, G). The RTA is pointed in *G. korowai* sp. nov. (Fig. 3) but blunt in *G. robusta* (Patoleta and Żabka 2020: figs 7, 8). The dco (dorsal cymbial outgrowth; Fig. 3E) is blunt and rounded.

The palps of *G. salax* and *G. korowai* sp. nov. are similar, as both RTAs are pointed. *Gelotia korowai* sp. nov. can be differentiated from *G. salax* by the length of the embolus: in *G. korowai* sp. nov. it reaches about the half the length of the bulb, whereas the embolus nearly reaches the RTA in *G. salax*. The RTA is 1.5× as long as the palpal tibia in *G. korowai* sp. nov. and as long as the tibia in *G. salax* (Wanless 1984: fig. 20C).

No females are known for *G. salax*. Females of *G. korowai* sp. nov. differ from those of *G. robusta* by the longer copulatory ducts (Fig. 4.), which are about half of the diameter of the spermathecae (about a third in *G. robusta*; Patoleta and Żabka 2020: fig. 17); the upward orientation of the fertilization ducts (Fig. 4C, D), which are directed downwards in *G. robusta* (Patoleta and Żabka 2020: fig. 17); and by the v-shaped sclerotized area around the copulatory organs, which are c-shaped in *G. robusta* (Patoleta and Żabka 2020: fig. 15).

##### Description.

**Figure 1.**
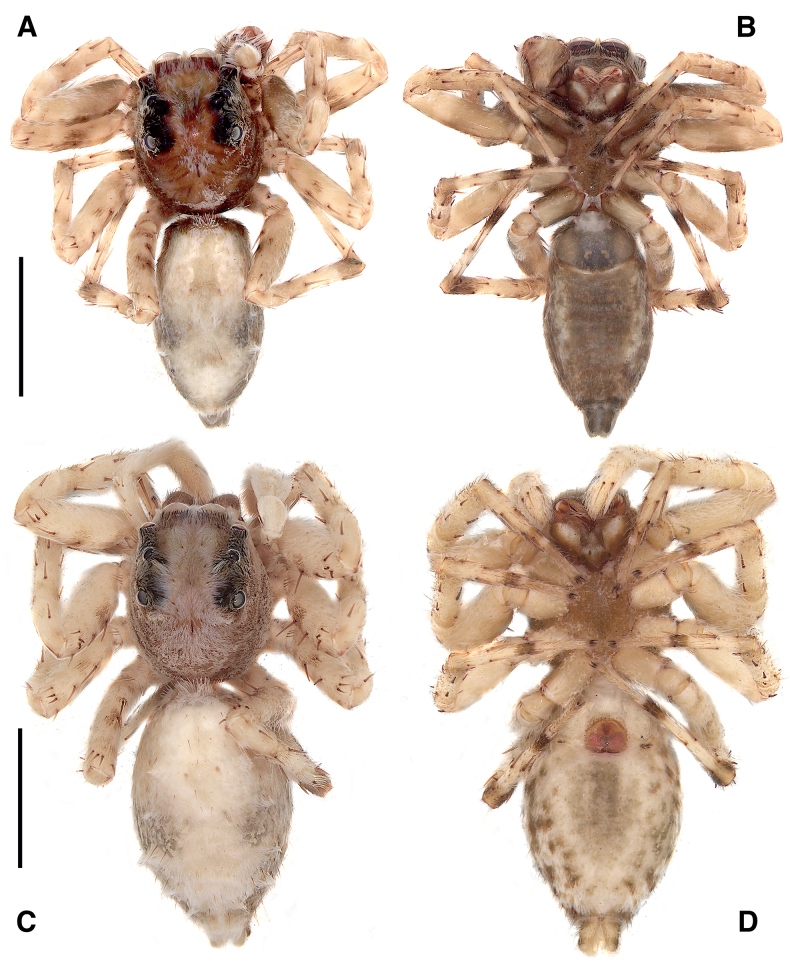
*Gelotia korowai* sp. nov., habitus. **A**. Holotype male, dorsal view; **B**. Ibid., ventral view; **C**. Paratype female, dorsal view; **D**. Ibid., ventral view. Scale bars: 2.0 mm.

**Figure 2.**
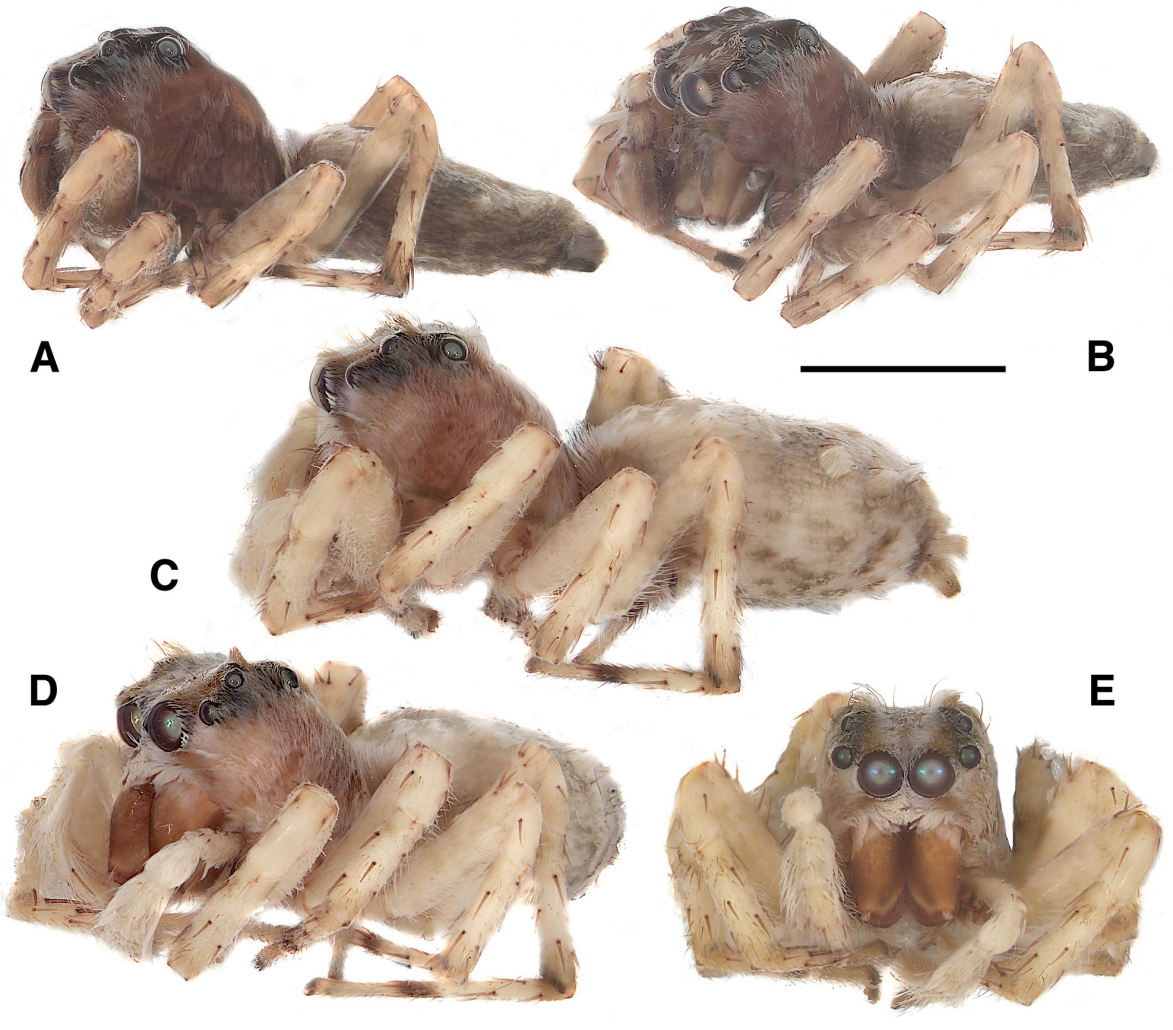
*Gelotia korowai* sp. nov., habitus. **A**. Holotype male, lateral view; **B**. Ibid., fronto-lateral view; **C**. Paratype female, lateral view; **D**. Ibid., fronto-lateral view; **E**. Ibid., frontal view. Scale bars: 2.0 mm.

**Figure 3.**
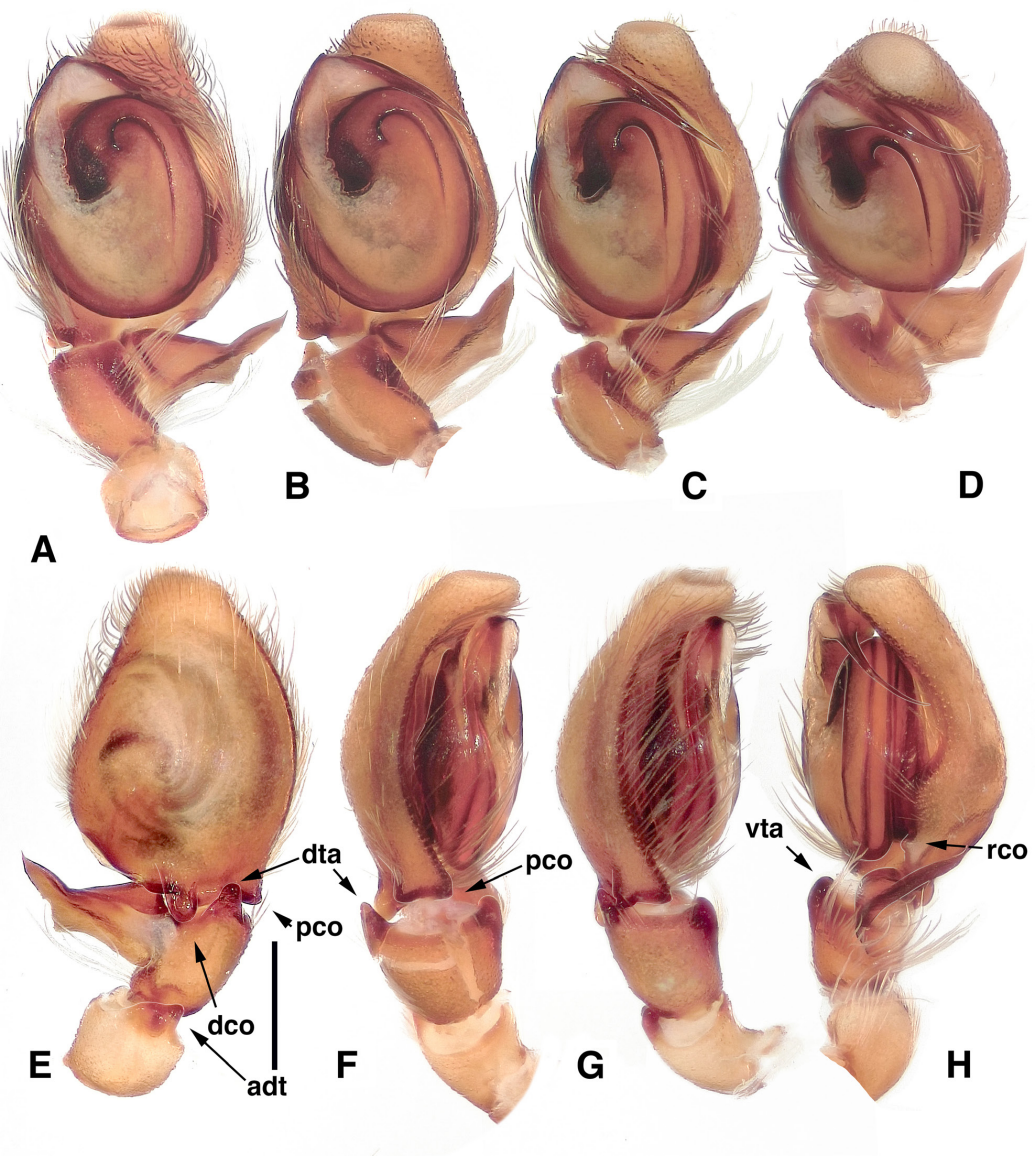
*Gelotia korowai* sp. nov., male palp. **A, E, G, H**. Holotype male; **B–D, F**. Paratype male; **A, B**. Ventral view; **C, D**. Latero-apical view; **E**. Dorsal view; **F, G**. Prolateral view; **H**. Retrolateral view. Abbreviations: adt: anteriodorsal tubercle, dco: dorsal cymbial process, pco: prolateral cymbial process, rco: retrolateral cymbial process, vta: ventral tibial apophysis. Scale bar: 0.4 mm.

**Male** (holotype). Habitus (Figs 1A, 1B, 2A, 2B). Total length 5.1. Carapace length 2.09, width 1.75. Abdomen length 3.01, width 1.42. Carapace round, brownish, with white/silver setae covering sides and thoracic slope (Fig. 1A). Ocular area covered in long, grey setae (Fig. 1A). Prosoma almost triangular in lateral view (Fig. 2A). Gnathocoxae and labium brown, with a white line in apical area (Fig. 1B). Chelicerae (Figs 1B, 2B) yellowish brown. Sternum oval, dark brown, covered with white setae (Fig. 1B). Opisthosoma longer than wide, dark brown, without any pattern (Figs 1A, 1B, 2A, 2B). Legs covered in fine white setae (Figs 1A, 1B, 2A, 2B). Leg measurements: I 5.07 II 5.06, III, 4.88, IV 5.81. Leg spination: Leg I: Fe d 1-1-4; Pa d 2; Ti d 1-0-1-1, pr and rt 1-1, v 2-2-2ap; Mt d 2-1-2, pr 1-1, rt 1-1-1, v 2. Leg II: Fe d 1-1-4; Pa d 2; Ti d 1-0-1-1, pr and rt 1-1, v 2-2-2ap; Mt d 2-1-2, pr 1-1, rt 1-1-1, v 2. Leg III: Fe d 1-1-4; Pa d 2; Ti d 1-0-1, pr and rt 1-1, v 2-2-2ap; Mt d 1-2, pr 1-1-1, rt 1-1-1, v 2-0-2. Leg IV: Fe d 1-1-4, Pa d 2; Ti d 1-0-1, pr and rt 1-0-1, v 2-2-2ap; Mt d 2-2-2, v 2-0-2. Palp (Fig. 3) dark brown, with round bulb, well-defined tegular furrow, and large RTA (Fig. 3). Palpal femur without apical apophysis; palp with an apical dorsal tubercle (adt) (Fig. 3E). Palpal tibia short, with a cap-like RTA, ‘dta’, and ‘vta’ (Fig. 3E, H). Cymbium with a pco, a dco, and an rco (Fig. 3E, H), with round bulb; tegular furrow long and curved (Fig. 3A–D). Embolus robust, relatively long, curving inwards towards alveolus (Fig. 3A–D, H). RTA large, triangular, with a slight curve on both upper and lower sides (Fig. 3A–E); dta small, tooth-like; vta similar to dta but larger (Fig. 3F–H). Cymbium with both dco and pco; both small and rounded (Fig. 3E).

**Figure 4.**
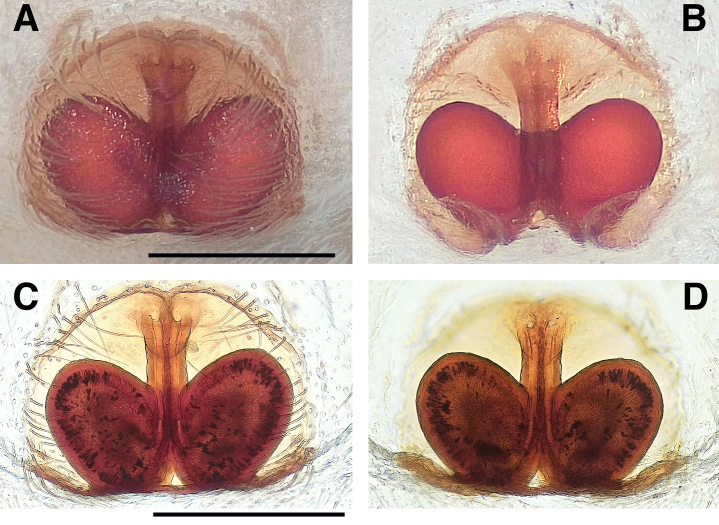
*Gelotia korowai* sp. nov., female copulatory organ. **A.** Intact epigyne, ventral view; **B.** Cleared epigyne, dorsal view; **C.** Vulva in methyl-salicylate, ventral view; **D.** Ibid., dorsal view. Scale bar: 0.4 mm.

**Female** (paratype). Habitus (Figs 1C, 1D, 2C–E). Total length 6.48. Carapace length 2.77, width 2.01. Abdomen length 3.71, width 2.39. Carapace brownish, covered in white setae (Figs 1C, 2C, 2D). Gnathocoxae and labium brown, with a white line in apical region (Fig. 1D). Chelicerae (Figs 1D, 2D, 2E) yellowish brown, with dense whitish setae (Figs 1D, 2D, 2E). Sternum oval, light brown, covered with setae (Fig. 1D). Legs covered in fine setae, with brown and white alternating patterns (Figs 1C, 1D, 2C–E). Leg measurements: I 5.36, II 5.39, III, 5.22, IV 6.33. Leg spination: Leg I: Fe d 1-1-4; Pa d 2; Ti d 1, pr and rt 1-0-1, v 2-2-2ap; Mt d 1-2, pr 1-1, rt 1-1, v 2. Leg II: Fe d 1-1-4; Pa d 2; Ti d 1, pr and rt 1-0-1, v 2-2-2ap; Mt d 1-2, pr 1-1, rt 1-1, v 2. Leg III: Fe d 1-1-4; Pa d 2; Ti d 1-0-1, pr 1-0-1, rt 1-1-1, v 2-2-2ap; Mt d 1-2, pr 1-1, rt 1-1, v 2-0-2. Leg IV: Fe d 1-1-4, Pa d 2; Ti d 1-0-1, pr 1-0-1 rt 1-1-1, v 2-2-2ap; Mt d 1-2, pr 1-1, rt 1-1, v 2-0-2. Entire body covered in fine, dense whiteish setae (Figs 1C, 1D, 2C, 2D, 2E). Opisthosoma round, pale, with brown patterns (Figs 1C, 1D, 2C–E). Spinnerets short (Figs 1C, 1D, 2D). Epigyne (Fig. 4) round, with median ridge and dark copulatory openings (Fig. 4). Spermathecae kidney-shaped, located near median ridge (Fig. 4). Sperm ducts highly folded, beneath spermathecae (Fig. 4C, D).

##### Distribution.

Known only from the type locality, Mt Missim (Fig. 10).

##### Natural history.

The male specimens were collected from trees of the genus *Lithocarpus* Blume; the holotype and one paratype were from the forest interior, and the other paratype was from the forest edge. The female specimen was found on a tree, *Castanopsis acuminatissima* (Blume) A.DC.

### Tribe Cocalodini Simon, 1901

#### Genus Cocalodes

Taxon classificationAnimaliaAraneaeSalticidae

Pocock, 1897

FBDA1F4C-D412-5F16-841F-AA8664F28E44

##### Type species.

*Cocalodes leptopus* Pocock, 1897 by original designation.

##### Diagnosis.

**Figure 5.**
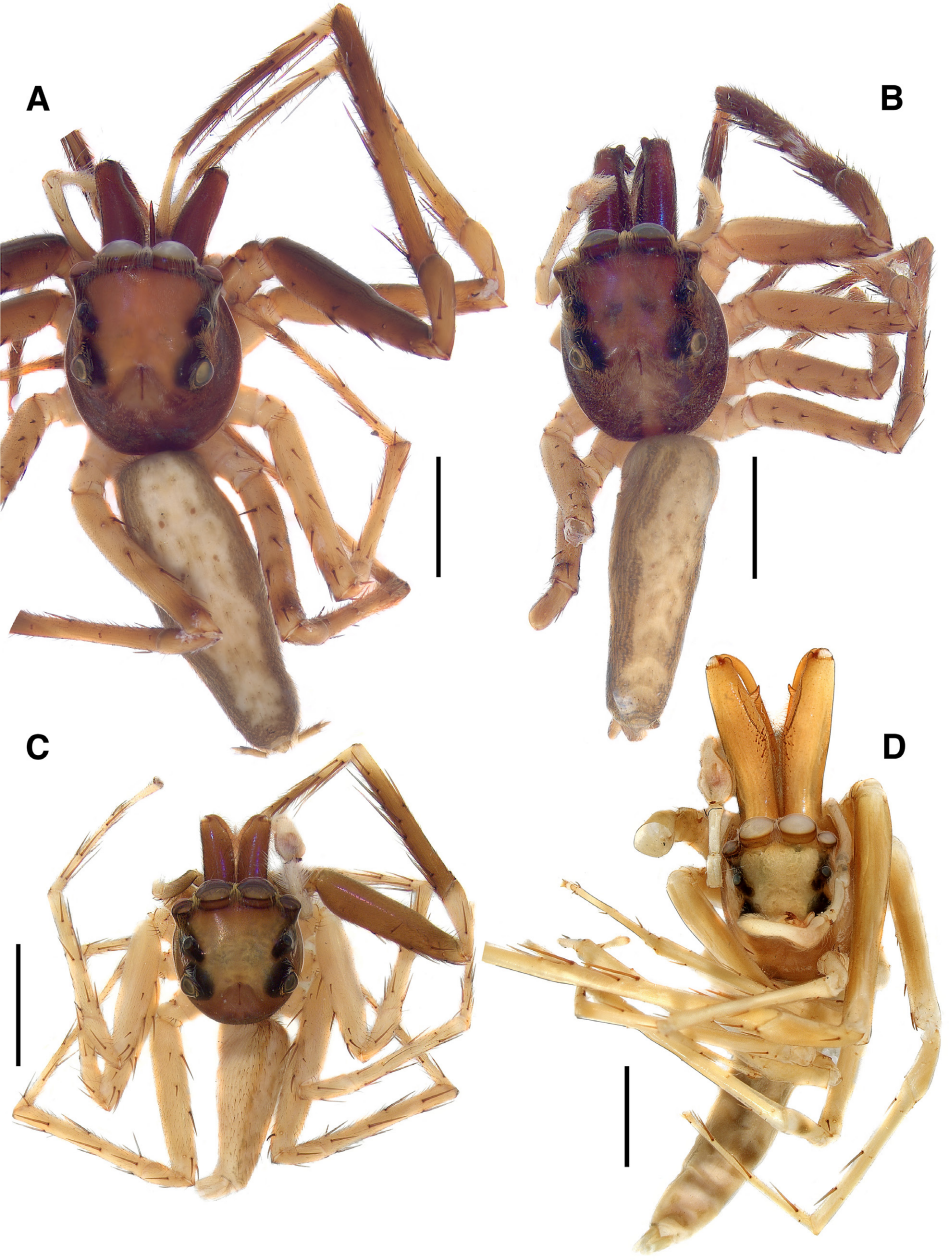
*Cocalodes* spp., habitus, males, dorsal views. **A**. *C. innotabilis* (HNHMAraneae-10722); **B**. *C. thoracicus* (HNHMAraneae-11642); **C**. *C. macellus* (HNHMAraneae-11674); **D**. *C. plebejus*, lectotype (HNHM-Araneae-4459). Scale bars: 2.0 mm.

**Figure 6.**
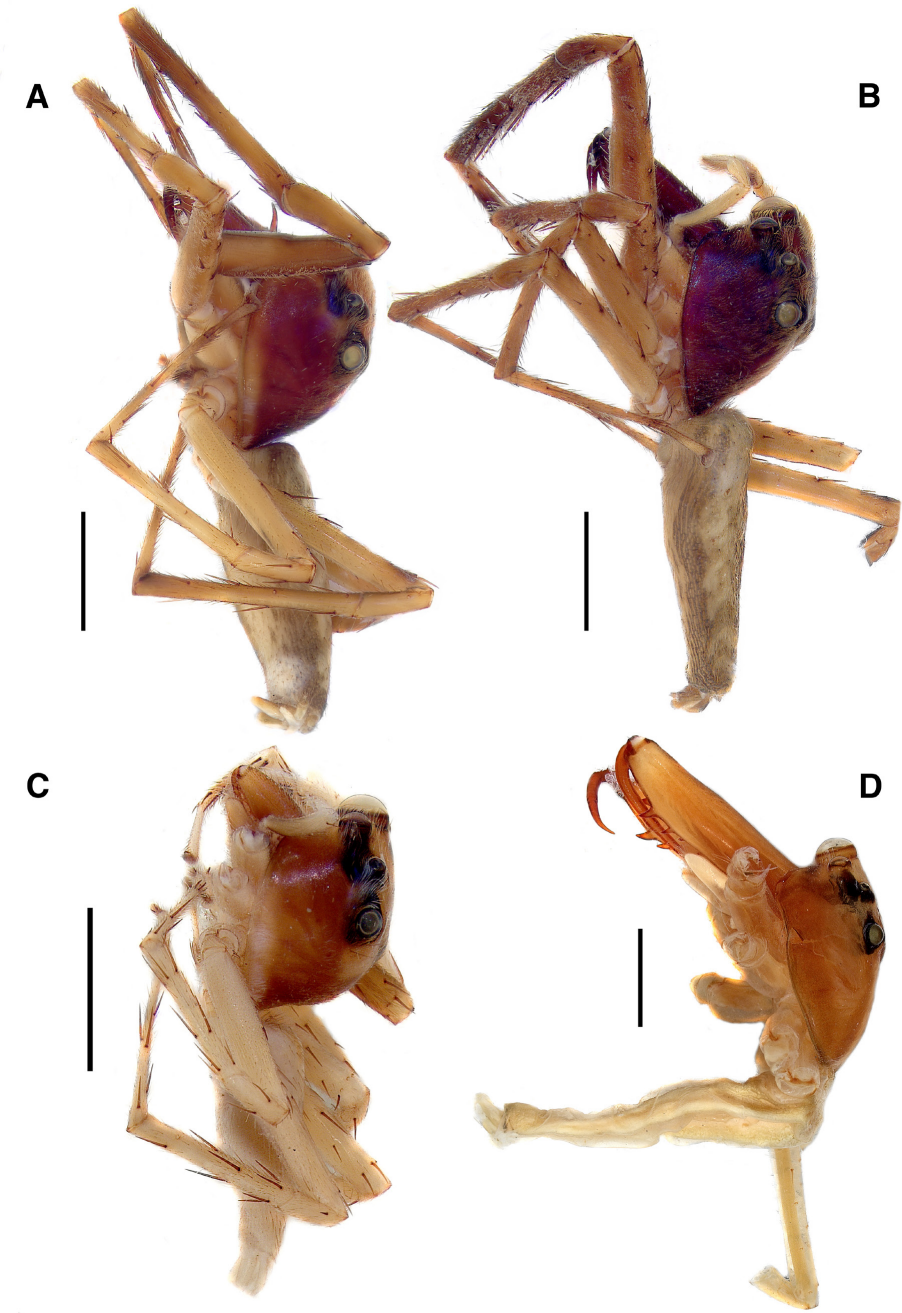
Habitus of *Cocalodes* spp., habitus, males, lateral view. **A**. *C. innotabilis* (HNHMAraneae-10722); **B**. *C. thoracicus* (HNHMAraneae-11642); **C**. *C. macellus* (HNHMAraneae-11674); **D**. *C. plebejus*, lectotype (HNHM-Araneae-7447). Scale bars: 2.0 mm.

**Figure 7.**
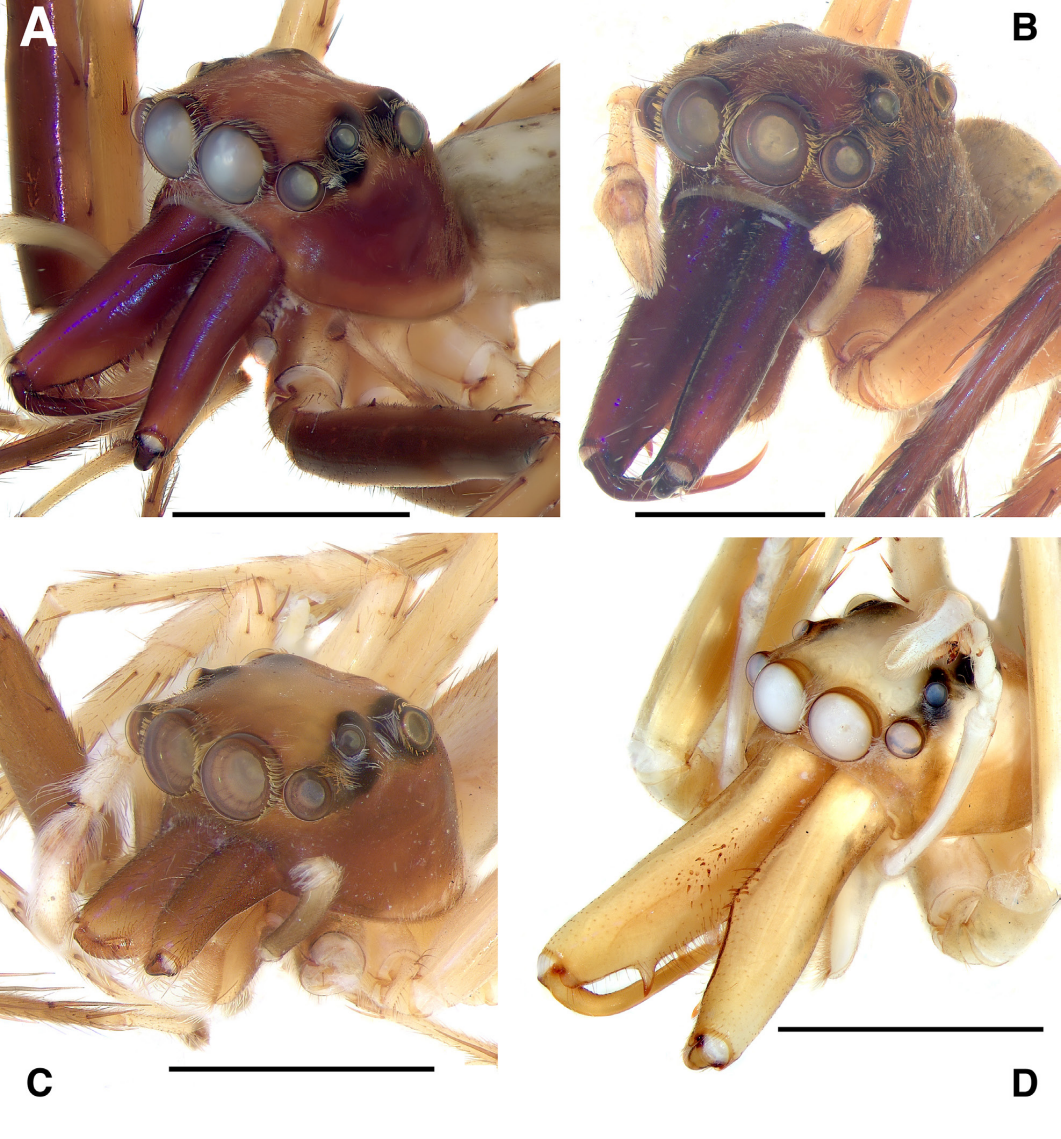
*Cocalodes* spp., prosoma, males, fronto-lateral view. **A**. *C. innotabilis* (HNHMAraneae-10722); **B**. *C. thoracicus* (HNHMAraneae-11642); **C**. *C. macellus* (HNHMAraneae-11674); **D**. *C. plebejus*, lectotype (HNHM-Araneae-4459). Scale bars 2.0 mm.

Males with more or less elongated chelicerae (Figs 5, 6, 7), often with enlarged intercheliceral sclerite horn. Male palp with an elongated femur and tibia. RTA small but well sclerotized; cymbium with a retrolateral proximal row of 3–5 peg-like setae (Fig. 9E–H, Wanless 1982: figs 1A, 2C, 4C, 6C, 7G, I, 10C, 15D, 18A). Upper half of cymbium elongated; bulb with a large apical conductor, a retrolateral median apophysis, and a filiform embolus originating from proximal part of the small tegulum.

##### Description.

For detailed description, see Wanless (1982).

##### Comments.

The genus has 12 described species from Indonesia and Papua New Guinea, and of those, 10 are known from the island of New Guinea. Four species are known only from one sex, and few new data have emerged since Wanless’s (1982) revision. Wanless (1982) delimited and illustrated all species well but was unable to examine two species, *Cocalodes plebejus* and *C. thoracicus*, described by Szombathy (1915).

#### Cocalodes innotabilis

Taxon classificationAnimaliaAraneaeSalticidae

Wanless, 1982

E3BCEE53-A0D4-5AA0-8E70-1AAD8DB77F6B

**Figure 8.**
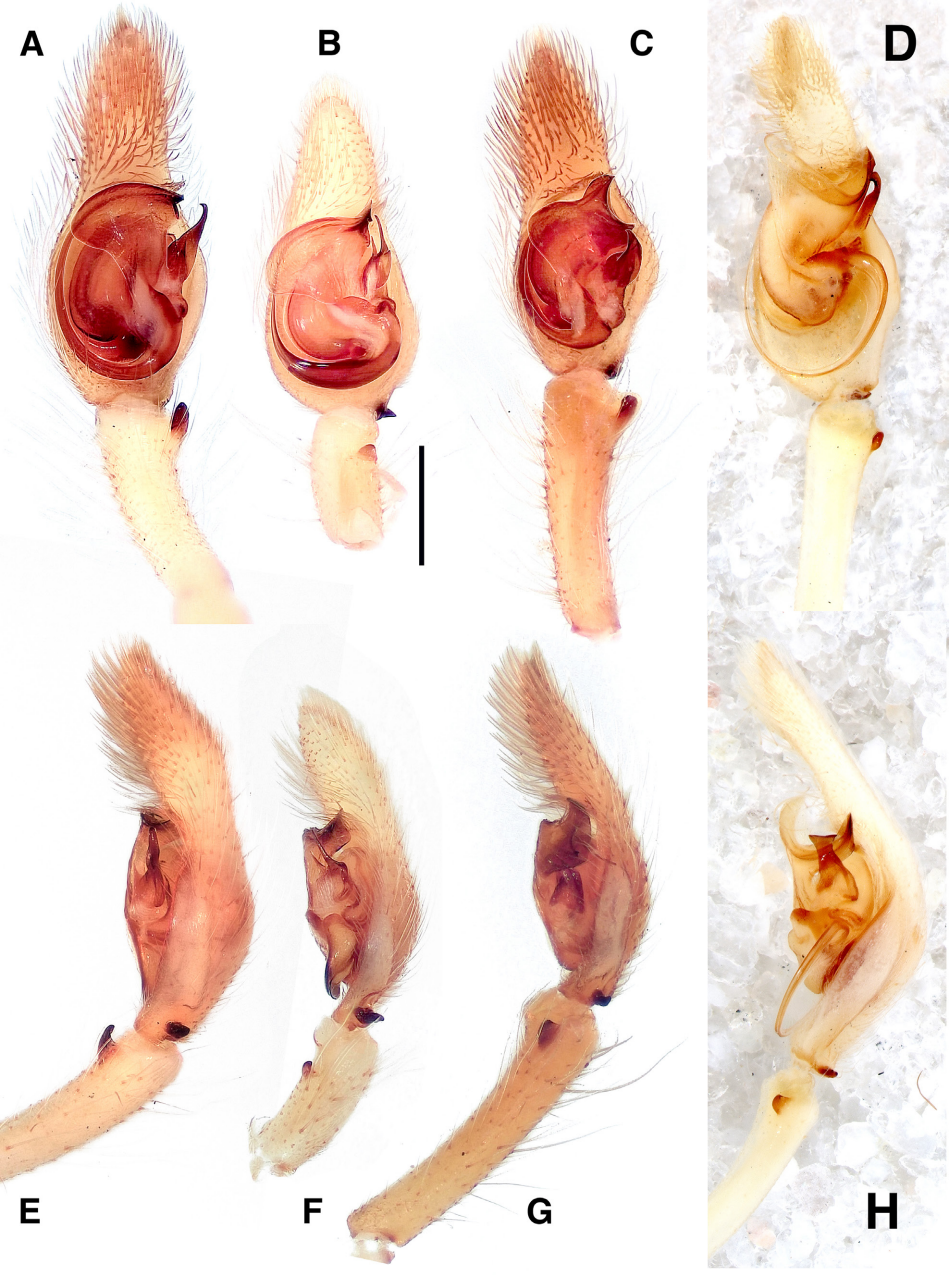
*Cocalodes* spp., male copulatory organs. **A–D**. Ventral view; **E–H**. Retrolateral view; **A, E**. *C. innotabilis* (HNHMAraneae-10722); **B, F**. *C. macellus* (HNHMAraneae-11674); **C, G**. *C. thoracicus* (HNHMAraneae-11642); **D, H**. *C. plebejus* lectotype (HNHM-Araneae-7447). Scale bars: 0.4 mm.

**Figure 9.**
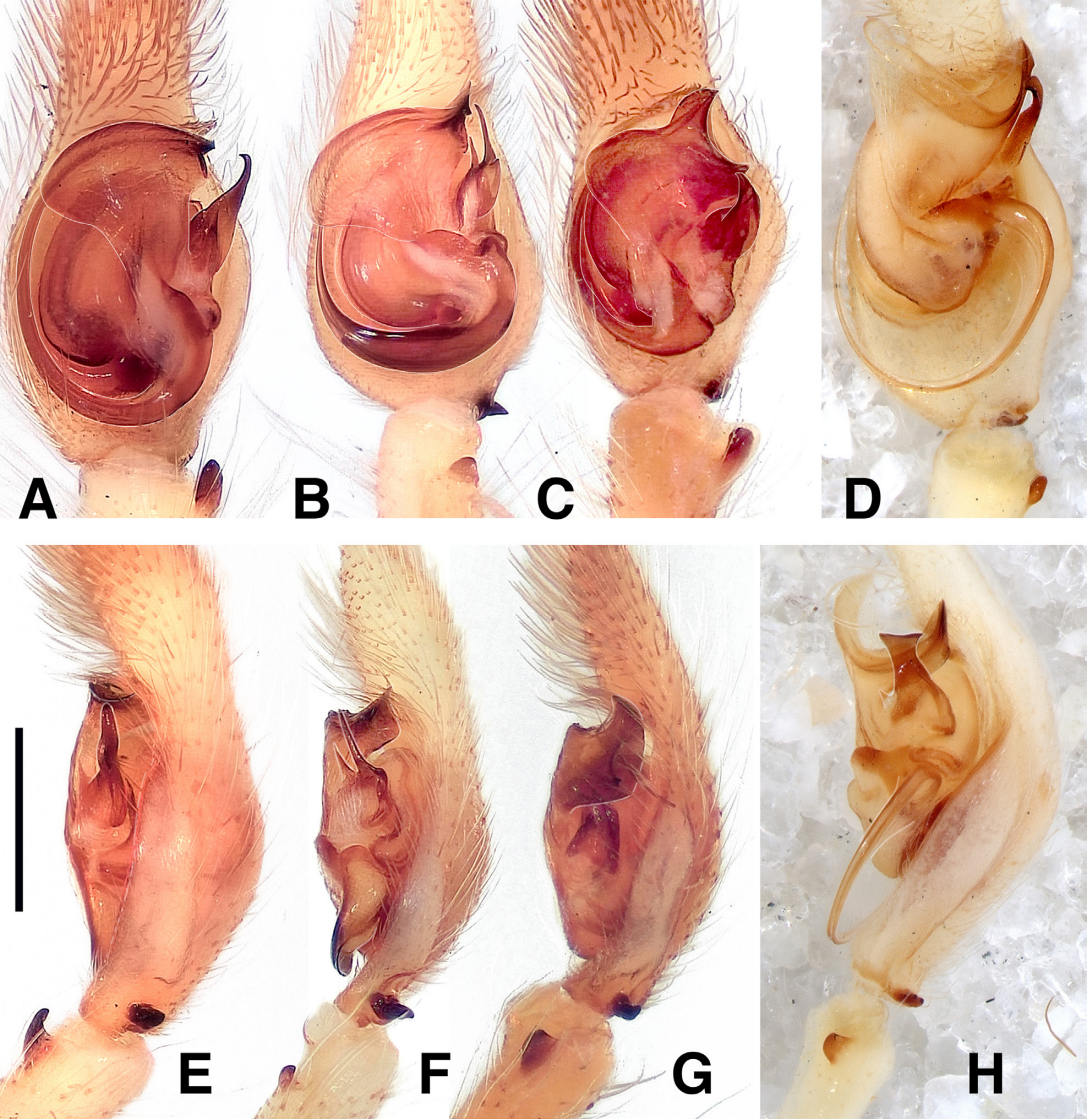
*Cocalodes* spp., male palpal bulbs. **A–D**. Ventral view; **E–H**. Retrolateral view; **A, E**. *C. innotabilis* (HNHMAraneae-10722); **B, F**. *C. macellus* (HNHMAraneae-11674); **C, G**. *C. thoracicus* (HNHMAraneae-11642). **D, H**. *C. plebejus* lectotype (HNHM-Araneae-7447). Scale bars: 0.4 mm.

Figs 5A, 6A, 7A, 8A, 8E, 9A, 9E

Cocalodes innotabilis Wanless 1982: 288.

##### Type material.

***Holotype* male** • Papua New Guinea; Louisiade Archipelago, Sudest Island: Rambuso, camp 11; 11°29.03'S, 153°32.47'E; 0–100 m a.s.l.; October 1956; leg. L.J. Brass and R.F. Peterson; Fifth Archbold Expedition to New Guinea; AMNH (not examined).

##### Other material examined.

Papua New Guinea • 1 male; Port Moresby Brown River; 9°12.37'S, 147°0.86'E; 5 m a.s.l.; 23 October 1960.; leg. J.L. Gressit; HNHM-Araneae 10722 • 3 males; same locality; 21 July 1969; leg. J. Balogh; HNHM-Araneae 11575, HNHM-Araneae 11683.

#### Cocalodes thoracicus

Taxon classificationAnimaliaAraneaeSalticidae

Szombathy, 1915

934175C3-FB26-5E1A-AFAE-78CE9263A91B

Figs 5B, 6B, 7B, 8C, 8G, 9C, 9G

Cocalodes thoracicus Szombathy 1915: 470.

##### Note.

For a detailed synonymy and citation list, see World Spider Catalog (2026).

##### Type material.

***Lectotype* male, here designated**: Papua New Guinea • Astrolabe Bay, Stephansort; 5°26.50'S, 145°45.34'E; 5 m a.s.l.; leg. L. Bíró; HNHM-Araneae 7449.

##### Other material.

Papua New Guinea • 1 male; Astrolabe Bay, Erima; 5°24.85'S, 145°43.94'E; 10 m a.s.l.; leg. L. Bíró; HNHM-Araneae 4464 • 1 male; Huon Peninsula, Simbang; 6°35.11'S, 147°49.21'E; 20 m a.s.l.; leg. L. Bíró; HNHM-Araneae 4462 • 1 male, 1 female; Astrolabe Bay, Stephansort; 5°26.77'S, 145°44.69'E; 15 m a.s.l.; leg. L. Bíró; HNHM-Araneae 4465 • 1 male, 1 juvenile; Madang; 5°14.79'S, 145°46.92'E; 30 m a.s.l.; leg. L. Bíró; HNHM-Araneae 4463 • 1 female; Mt Missim, 6 km N, 7 km of W Wau Aerodrome; 7°17.28'S, 146°39.12'E; 1350 m a.s.l.; 25 August 1988; leg. A. Allison; Tree 18 Tray 9; BM • 1 male, 1 female from Mt Kaindi; 7°20.10'S, 146 40.91'E; 30 m a.s.l.; leg. J. Balogh; HNHM-Araneae 11642.

##### Remarks.

As mentioned by Wanless (1982), the types were in “Termeszettudomanyi Muzeum, Budapest”, but unfortunately it was impossible for him to examine them. We were able to locate five vials labelled *Cocalodes thoracicus*, HNHM-Araneae 4462 to 4465 and 7449. The localities of these specimens match those in the original description (Szombathy 1915: 472): “Erima, Friedrich-Wilhelmshafen [now Stephansort] (baie de l’Astrolabe), Simbang (Huon golf)”. We designate a lectotype for *C. thoracicus* here.

Szombathy (Szombathy 1915) did not illustrate the male palp nor the epigyne, but the chelicera has a unique lobe that allowed Wanless (1982) to recognize the species and illustrate its copulatory organs. When examining the lectotype male, we confirmed Wanless’s (1982) identification. For a detailed description, see Wanless (1982).

#### Cocalodes macellus

Taxon classificationAnimaliaAraneaeSalticidae

Thorell, 1878

66F41C44-7C16-5B49-AF5D-662194659BB2

Figs 5C, 6C, 7C, 8B, 8F, 9B, 9F

Cocalus macellus Thorell 1878: 287.

##### Note.

For a detailed synonymy and citation list, see World Spider Catalog (2026).

##### Type material.

• 1 female; Indonesia; Maluku, Ambon; 3°39.92'S, 128°4.79'E; 30 m a.s.l.; leg. Beccari; MCSN, Genova (not examined).

##### Material examined.

Papua New Guinea • 2 males; Lae; 6°40.58'S, 146°54.53'E; 30 m a.s.l.; leg. J. Balogh; HNHM-Araneae 11601, HNHM-Araneae 11674.

##### Remarks.

Wanless (1982) supplied excellent drawings of the male palp and habitus, as well as the female copulatory organs. Maddison (2009) provided additional data on this species from Tualapa and figured living specimens of both sexes. For detailed description, see Wanless (1982).

#### Cocalodes papuanus

Taxon classificationAnimaliaAraneaeSalticidae

Simon, 1900

97635ECD-2A86-5266-B506-F6667E0C0D3B

Figs 5D, 6D, 7D, 8D, H, 9D, H

Cocalodes papuanus Simon 1900: 32.
Cocalodes armatissimus Strand 1913: 122.
Cocalodes plebejus Szombathy 1915: 468.

##### Note.

For a detailed synonymy and citation list, see World Spider Catalog (2026).

##### Type material.

*C. papuanus*, lectotype male; Indonesia • Irian Jaya; MNHN (not examined). *C. armatissimus*: lectotype male; Papua New Guinea • Schouten Islands, Vokeo; FS (not examined). *C. plebejus*: ***lectotype* male designated here**; Indonesia • Madura Island; 7°1.37'S, 113°18.71'E; 100 m a.s.l.; leg. L. Bíró; HNHM-Araneae 4459.

##### Other material examined.

Papua New Guinea • 1 male; Garove Island; 4°39.25'S, 149°30.36'E; 100 m a.s.l.; 1900; leg. L. Bíró; HNHM-Araneae 7447 • 1 female; same locality; HNHM-Araneae 7448 • 3 juveniles; Astrolabe Bay, Stephansort; 5°26.50'S, 145°45.34'E; 5 m a.s.l.; leg. L. Bíró; HNHM-Araneae 4460 • 1 female, 1 juvenile; Tumleo Island; 3°7.19'S, 142°23.83'E; leg. L. Bíró; HNHM-Araneae 4461.

##### Remarks.

As mentioned by Wanless (1982), the Szombathy’s type material of *C. plebejus* is “presumably in TM, Budapest”, and we were able to locate the specimens in the HNHM. There are 10 vials in that collection identified as *Cocalodes*. Five of them are labelled as *C. thoracicus*, and five as “*Cocalodes communis*”. However, there is no known species with the communis epithet, and *C. plebejus* was not used on any labels in the HNHM collection. Because the Latin words plebejus and communis have similar meanings, one of these tubes is labelled as the “typus”, and the localities on the labels match those in the original description—”India or Madura, Stephansort (Astrolabe Bay), Tamara (Berlinhafen), I. Declacs [sic!]” (Szombathy 1915: 470)—we can assume that these five tubes contain the syntypes of *C. plebejus*. These tubes are HNHM-Araneae 4459 to 4461, 7447, and 7448. There is some inconsistency in the listed specimens (“3 mâles, 3 femelles, 4 immatures”) and what we found in the collection (2 males, 2 females, 4 juveniles).

Although there is a tube in the collection with a male in poor condition (Fig. 6D; both palps are detached, Figs 8D, 8H) with the label “typus”, Szombathy (1915) neither selected a type specimen nor distinguished a specimen as the holotype in the original description of *C. plebejus*. Thus, all these specimens are considered syntypes. Although *C. plebejus* is a junior synonym, due to the labelling differences and for taxonomic stability, we here designate a lectotype. We have chosen HNHM-Araneae 4459, a male with both palps still attached (Figs 5D, 7D).

**Figure 10.**
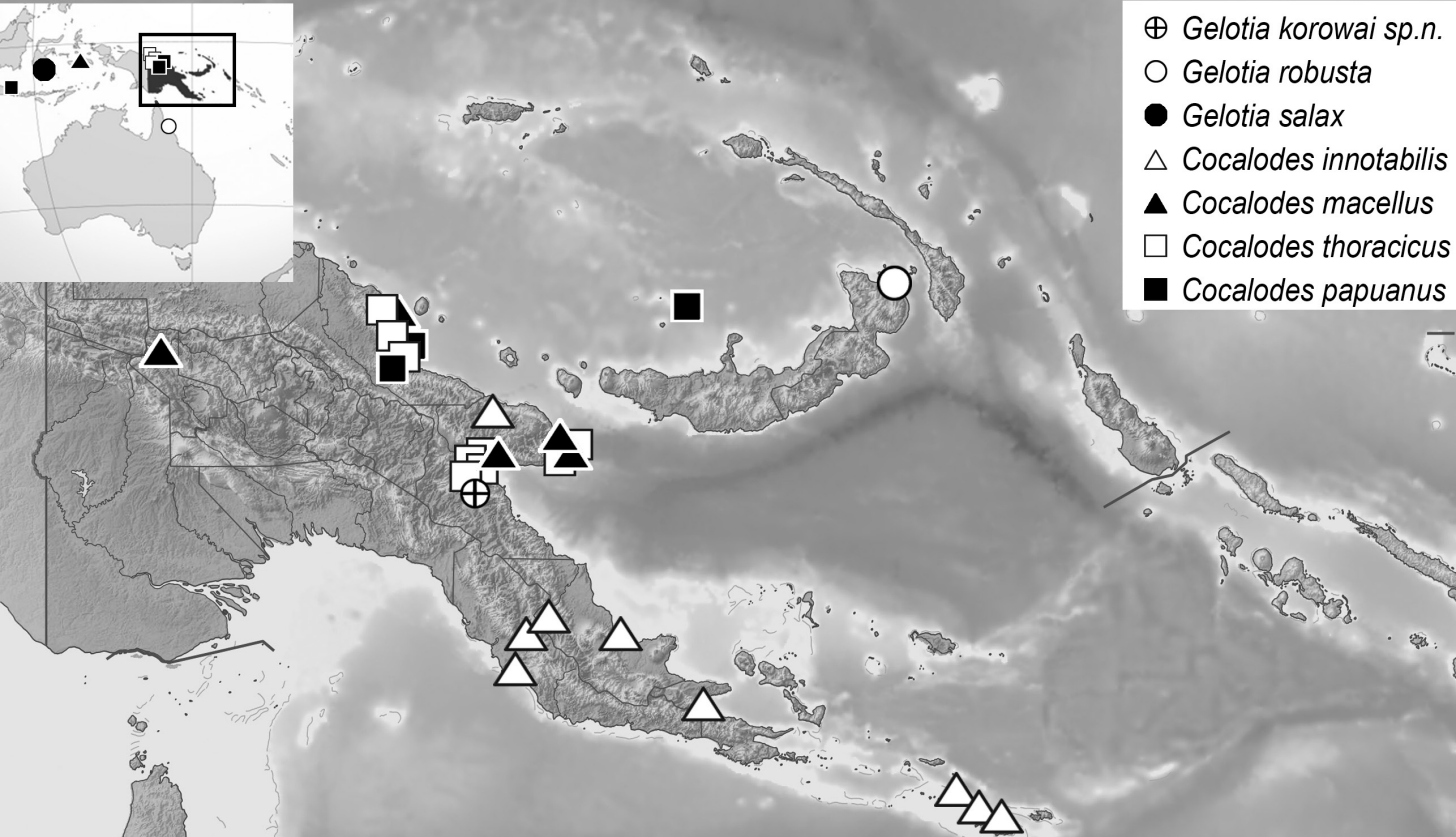
Distribution map of Cocalodes and Gelotia species discussed.

The above synonymy proposed by Wanless is supported by detailed figures of the palp and habitus provided by Szombathy (1915) and Wanless (1982). The species has been found not only in Papua New Guinea (Vokeo Island, Madang) but in Indonesia (Manokwari Bay, Jayapura). Maddison et al. (2014) illustrated a male from Baiteta Forest (Madang). The species possesses a unique cheliceral formation and therefore is possible to identify from photographs. However, it has not been observed on iNaturalist or similar citizen-scientist platforms.

For a detailed description see Wanless (1982).

## Supplementary Material

XML Treatment for Gelotia

XML Treatment for Gelotia korowai

XML Treatment for Cocalodes

XML Treatment for Cocalodes innotabilis

XML Treatment for Cocalodes thoracicus

XML Treatment for Cocalodes macellus

XML Treatment for Cocalodes papuanus

## Additional information

### Artificial Intelligence (AI) use

The authors accept full responsibility for the content of the manuscript, including the disclosure of any use of AI.

No AI tools were used in the preparation of this manuscript.

### Author ORCIDs

H. Takács-Vágó https://orcid.org/0009-0003-7837-1979

T. Szűts https://orcid.org/0000-0001-8954-0641

K. Szabó https://orcid.org/0000-0002-5110-8279

### Data availability

All of the data that support the findings of this study are available in the main text.
